# Blood-based epigenome-wide association study and prediction of alcohol consumption

**DOI:** 10.1186/s13148-025-01818-y

**Published:** 2025-01-25

**Authors:** Elena Bernabeu, Aleksandra D. Chybowska, Jacob K. Kresovich, Matthew Suderman, Daniel L. McCartney, Robert F. Hillary, Janie Corley, Maria Del C. Valdés-Hernández, Susana Muñoz Maniega, Mark E. Bastin, Joanna M. Wardlaw, Zongli Xu, Dale P. Sandler, Archie Campbell, Sarah E. Harris, Andrew M. McIntosh, Jack A. Taylor, Paul Yousefi, Simon R. Cox, Kathryn L. Evans, Matthew R. Robinson, Catalina A. Vallejos, Riccardo E. Marioni

**Affiliations:** 1 Centre for Genomic and Experimental Medicine, Institute of Genetics and Cancer, University of Edinburgh, Edinburgh, UK; 2 Department of Cancer Epidemiology, H. Lee Moffitt Cancer Center and Research Institute, Tampa, FL USA; 3 Medical Research Council Integrative Epidemiology Unit, University of Bristol, Bristol, BS8 1TH UK; 4 Edinburgh Medical School, Usher Institute, University of Edinburgh, Edinburgh, UK; 5 Lothian Birth Cohorts, Department of Psychology, University of Edinburgh, Edinburgh, UK; 6 Scottish Imaging Network, A Platform for Scientific Excellence (SINAPSE) Collaboration, Edinburgh, UK; 7 Centre for Clinical Brain Sciences, Edinburgh Imaging and UK Dementia Research Institute, University of Edinburgh, Edinburgh, UK; 8 Neurovascular Imaging Research Core, UCLA, Los Angeles, CA USA; 9 Epidemiology Branch, National Institute of Environmental Health Sciences, Research Triangle Park, NC USA; 10 Division of Psychiatry, Royal Edinburgh Hospital, University of Edinburgh, Edinburgh, UK; 11 Institute of Science and Technology Austria, Klosterneuburg, Austria; 12 Medical Research Council Human Genetics Unit, Institute of Genetics and Cancer, University of Edinburgh, Edinburgh, UK; 13 The Alan Turing Institute, London, UK

## Abstract

Alcohol consumption is an important risk factor for multiple diseases. It is typically assessed via self-report, which is open to measurement error through recall bias. Instead, molecular data such as blood-based DNA methylation (DNAm) could be used to derive a more objective measure of alcohol consumption by incorporating information from cytosine-phosphate-guanine (CpG) sites known to be linked to the trait. Here, we explore the epigenetic architecture of self-reported weekly units of alcohol consumption in the Generation Scotland study. We first create a blood-based epigenetic score (EpiScore) of alcohol consumption using elastic net penalized linear regression. We explore the effect of pre-filtering for CpG features ahead of elastic net, as well as differential patterns by sex and by units consumed in the last week relative to an average week. The final EpiScore was trained on 16,717 individuals and tested in four external cohorts: the Lothian Birth Cohorts (LBC) of 1921 and 1936, the Sister Study, and the Avon Longitudinal Study of Parents and Children (total N across studies > 10,000). The maximum Pearson correlation between the EpiScore and self-reported alcohol consumption within cohort ranged from 0.41 to 0.53. In LBC1936, higher EpiScore levels had significant associations with poorer global brain imaging metrics, whereas self-reported alcohol consumption did not. Finally, we identified two novel CpG loci via a Bayesian penalized regression epigenome-wide association study of alcohol consumption. Together, these findings show how DNAm can objectively characterize patterns of alcohol consumption that associate with brain health, unlike self-reported estimates.

**Supplementary Information:**

The online version contains supplementary material available at 10.1186/s13148-025-01818-y.

## Introduction

Alcohol consumption, particularly heavy use, has been associated with increased morbidity and mortality, cognitive impairment, progressive white matter degeneration in the brain, and is a major risk factor for various forms of cancer [1–5]. Despite regular alcohol use being an important risk factor for a plethora of diseases, self-reported consumption is an imperfect phenotype that can be prone to recall bias [6].

Like other environmental and lifestyle factors [7, 8], alcohol consumption is linked to the epigenome, specifically blood-based DNA methylation (DNAm) patterns [9–11]. DNAm is an epigenetic mark that is typically characterized by the addition of a methyl group to the 5’ carbon of a cytosine base, often occurring at cytosine-phosphate-guanine (CpG) dinucleotides, also referred to as a CpG site [12]. DNAm can influence gene expression and cellular function; thus, methylomic modifications could mediate alcohol–disease risk associations and development [12, 13] as well as alcohol addiction [14, 15]. As such, identification of alcohol-associated CpG sites could provide biological insights into the pathophysiology of alcohol-related diseases [11, 16].

DNAm-based predictors of complex traits have gained prominence in recent years through the prediction of phenotypes such as age and smoking [8, 11, 17, 18]. The previous largest epigenome-wide association (EWAS) meta-analysis study of alcohol consumption (self-reported units consumed per day in the past year) included over 13,000 individuals from 13 cohorts. Using a 144-CpG signature, the authors explained up to 13.8% of the variance in the phenotype (incremental *R*^2^ over linear regression models including age and sex) in four independent test sets. There are two reasons why a DNAm-based predictor might provide an improved index of alcohol consumption. First, similar to the way that hair cortisol and glycated haemoglobin track long-term stress and glucose regulation, the methylome, although dynamic, is relatively stable. For example, many CpG sites associated with smoking revert back to similar levels as non-smokers around 5 years after quitting [7]. However, some sites remain differentially methylated up to 30 years after cessation [19]. Second, under the assumption that enough individuals respond accurately in a self-report questionnaire and by averaging over multiple CpG sites to build a predictor, one should be able to gain more precise estimates for those whose self-report data are inaccurate.

In this study, we explore the creation of an epigenetic predictor of alcohol consumption, making use of a large single-cohort DNAm study, Generation Scotland. We assess the performance of this predictor in 9 independent external subsets from four different studies: older adults across the 8th and 9th decades of life—the Lothian Birth Cohorts (LBC) of 1921 and 1936 [20, 21]; adolescents and adults from the Avon Longitudinal Study of Parents and Children (ALSPAC) [22, 23]; and multi-ancestry adult women from the Sister Study [24]. We also explore differential patterns by sex and units consumed in the last week relative to an average week. Furthermore, to gain further biological insights into potential alcohol-mediated pathways underlying disease, we perform the largest epigenome-wide association study (EWAS) of alcohol consumption to date (*N* = 16,717).

## Results

A total of 16,717 Generation Scotland participants (mean age 47.5, SD 14.9 years; 9758 females and 6959 males) had blood-based DNAm (see “Methods”) and self-reported alcohol consumption data available (Table 1, Supplementary Table 1, Supplementary Fig. 1). The mean alcohol units (unit definition as per UK National Health Service definition at 8 g/10 ml of pure alcohol) consumed in the week prior to completing the questionnaire and blood draw was 10.9 (SD 12.7, Supplementary Figs. 2 and 3). A total of 10,506 (62.8%) participants reported that this number was reflective of their usual drinking pattern (“normal week” drinkers) with 1622 and 3756 noting it was less or more than they typically drink in a week, respectively (response unknown for *N* = 833).

**Table 1:** Generation Scotland cohort classification by units drunk per week (unit definition as per UK National Health Service definition at 8 g/10 ml of pure alcohol), for both people reporting usual drinking (“normal week” drinkers) and everyone in the cohort

	Everyone		“Normal week” drinkers	
Category	*N*	*N* Females (%)	*N*	*N* Females (%)
Non-drinkers	3192	2313 (72.46)	1874	1396 (74.49)
Light/moderate drinkers	10,284	6085 (59.17)	6712	3918 (58.37)
Moderate/heavy drinkers	3241	1361 (42)	1920	777 (40.47)
Total	16,717	9759 (58.38)	10,506	6091 (57.98)

Moderate/heavy drinker threshold is 14 units per week for females and 21 for males

### Alcohol consumption EpiScore

An epigenetic score (EpiScore) was trained on self-reported alcohol units (log(x + 1) transformed) consumed in the week prior to the blood draw for DNAm measurement. Generation Scotland was split into a training (*N* = 8684) and test set (*N* = 8033).

We evaluated whether pre-selecting CpGs ahead of training could improve prediction performance. We trained predictors on either the full methylome (386,399 CpGs after limiting measured features to those also present in the Illumina 450 K array for wider applicability) or 3999 CpGs with evidence of an association to alcohol consumption in three recent EWASs that excluded Generation Scotland [9, 16, 25] (see Methods). Additionally, we evaluated whether training on a subset of 5618 (69.9% of the training set) individuals who reported that their consumption in the previous week was reflective of a “normal week” influenced predictor performance.

EpiScore prediction performance was assessed by Pearson correlations (*r*) between self-reported alcohol consumption units per week and the EpiScore, as well as by calculating the incremental *R*^2^ upon the addition of the EpiScore to a linear regression model adjusting for age and sex in the test set. We found that predictors trained on pre-filtered CpGs ahead of elastic net outperformed those trained on all CpG. We also found no improvement when training on individuals whose consumption in the previous week was reflective of a normal week (Table 2, Supplementary Fig. 4) possibly due to a reduced sample size (69.9% of the full set).

**Table 2:** Predictive performance and number of features of four EpiScores generated using elastic net regression

			Everyone		Normal week		More than normal		Less than normal	
			(*N* = 8033)		(*N* = 4888)		(*N* = 1920)		(*N* = 834)	
Training	CpGs	Elastic net N selected features	*r*	*R*^2^	*r*	*R*^2^	*r*	*R*^2^	*r*	*R*^2^
Everyone (*N* = 8684)	All	551	0.42	16.2	0.47	20.1	0.38	13.6	0.31	9.6
	Pre-selected	343	0.44	18.2	0.5	22.6	0.4	15.4	0.31	9.6
“Normal week” Drinkers (*N* = 5618)	All	460	0.4	15.3	0.45	19	0.36	13.4	0.3	9.2
	Pre-selected	360	0.42	16.9	0.48	21.6	0.39	14.8	0.29	8.7

Predictive performance was assessed in a holdout subset (test set) of Generation Scotland via Pearson correlations (r) and incremental R^2^ upon the addition of the EpiScore to a linear regression model for log alcohol units (+1) adjusting for age and sex. Alcohol units defined as per NHS guidelines (8 g/10 ml of pure alcohol). All EpiScores were marginally associated with self-reported alcohol consumption (*P *< 2.2 × 10^–16^).* P*-values taken for the EpiScore from the age- and sex-adjusted linear regression model

If the methylome is only able to capture recent exposure to alcohol, then our predictors should showcase differential performance if a person had deviated from their normal alcohol units consumed in a given week (drinking more or less than normal). We therefore evaluated EpiScore performance on participants who reported their alcohol consumption was similar to a normal week versus those reporting having consumed more or less than normal over the past week (N = 7642/8033, 95.1% of the testing dataset—status not recorded for 391 individuals). This consisted of 4888 individuals whose consumption in the past week was reflective of normal drinking behaviour, 1920 people who drank more than usual that week, and 834 people who drank less than usual. The EpiScore performed best in the subset of the test set that reported their consumption in the last week to be reflective of a normal week (Table 2, Supplementary Fig. 5).

### Alcohol EpiScore tested in external cohorts

The previous analyses established that model training was optimized by: (1) considering everyone and not just “normal week” drinkers and (2) pre-filtering to CpGs previously associated with alcohol. We therefore trained a final model in this manner making use of the full Generation Scotland cohort (*N* = 16,717). This returned an EpiScore consisting of 659 features (Supplementary Table 2). Predictive performance was evaluated in four external cohorts: the Lothian Birth Cohorts (LBC) of 1921 and 1936 (N = 436 and 895, respectively); ALSPAC (5 cohort subsets, N_TOTAL_ = 4083, ranging from 476 to 1482 per subset); and the Sister Study cohorts (2 cohort subsets, N_TOTAL_ = 5119, with N = 2770 and 2349 per subset, see Methods, Table 3). The LBC and ALSPAC cohorts reported alcohol consumption as average units consumed in a week the year prior to sampling (using the NHS unit definition of 8 g/10 ml of pure alcohol), while the Sister Study cohorts reported a derived variable that represented the average number of drinks per week over the last year. For simplicity, here we use the term “units per week” for all cohorts. All alcohol measurements were log(x + 1) transformed.

**Table 3:** EpiScore performance metrics in the Lothian Birth Cohorts, ALSPAC, and Sister Study cohorts

	Cohort	N	Age (mean, (SD))	N Males (%)	Alcohol UPW (median, [IQR])	r	R^2^
LBC	LBC1921	436	79.1 (0.6)	173 (39.7)	1.0 [0.5, 7.0]	0.41	17.9
	LBC1936	895	69.6 (0.8)	453 (50.6)	5.0 [0.5, 14.0]	0.42	16.6
ALSPAC	15up 450 K	670	17.4 (0.9)	311 (46.4)	3.0 [1.6, 6.9]	0.11	1.3
	15up EPIC	1482	17.8 (0.4)	704 (47.5)	4.3 [1.3, 6.9]	0.11	1.2
	F24	804	24.4 (0.8)	409 (50.9)	4.7 [2.3, 10.0]	0.30	9.3
	FOF	476	53.3 (5.3)	476 (100.0)	6.9 [3.0, 10.0]	0.45	20
	FOM	651	47.4 (4.4)	0 (0.0)	4.3 [1.3, 6.9]	0.33	10.7
SS	450 K	2770	57.0 (8.8)	0 (0.0)	1.0 [0.0, 4.0]	0.53	28.1
	EPIC	2349	55.5 (8.7)	0 (0.0)	0.5 [0.0, 3.0]	0.51	26.9
	EPIC nH White	1553	56.4 (8.8)	0 (0.0)	0.9 [0.1, 4.0]	0.51	26.6
	EPIC Black	796	53.7 (8.3)	0 (0.0)	0.1 [0.0, 1.0]	0.37	14.4

Model trained on all of Generation Scotland (N = 16,717, mean age = 47.3 (SD = 14.82), % males = 41.63%, mean alcohol consumption units (8 g/10 ml of pure alcohol) per week = 10.9 (SD = 12.72)). Cohort demographic metrics, as well as EpiScore performance metrics (r and incremental R^2^ over model adjusting for age and sex), are shown. LBC = Lothian Birth Cohort; SS = Sister Study; ALSPAC = Avon Longitudinal Study of Adults and Children; 15up = 15–17-year-olds, measured either in 450 K or EPIC arrays; F24 = 24-year-olds; FOF: fathers in midlife; FOM: mothers in midlife; UPW: units per week (NHS definition is 8 g/10 ml of pure alcohol)

In the two Scottish LBC studies (Supplementary Table 3), the EpiScore had a moderate correlation with self-reported alcohol consumption (r_LBC21_ = 0.41, r_LBC36_ = 0.42). The EpiScore had an incremental R^2^ (over a linear regression model adjusting for age and sex) of 17.9% in LBC1921 and of 16.6% in LBC1936 (Table 3, Supplementary Fig. 6). This outperforms a previously published alcohol consumption EpiScore trained on a subset of N = 2819 in Generation Scotland [8], which, when retested here to ensure equal LBC testing data pre-processing (the original paper tested on alcohol units as opposed to log(units + 1)), presented an incremental *R*^2^ over a model adjusting for age and sex of 6.3% in LBC1921 and 10.6% in LBC1936.

Considering the five ALSPAC cohort subsets (15up 450/EPIC: 15–17-year-olds measured on either 450 K or EPIC Illumina chips, F24: 24-year-olds, FOM: mothers in midlife, and FOF: fathers in midlife), the EpiScore correlation with self-reported alcohol consumption ranged from *r* = 0.11 to 0.45, with an incremental *R*^2^ over a linear regression model adjusting for age and sex ranging from 1.2 to 20%. Notably, the worst performing subsets were made up of younger individuals (mean age less < 18). The two Sister Study cohort subsets (one measured with 450 K array and another with EPIC array) showed correlations of *r* = 0.53 and 0.51, and with an incremental *R*^2^ = 28.1% and 26.9%, respectively (Table 3, Supplementary Figs. 7 and 8). The mean age in the Sister Study cohorts was approximately 56 years old. In a subset of 796 individuals of the Sister Study, who self-identified as Black, the correlation and incremental *R*^2^ were 0.37 and 14.4%, respectively,

### Measured alcohol consumption and EpiScore associations

We next tested for associations between self-reported alcohol consumption or our alcohol EpiScore and a number of lifestyle/health/socioeconomic factors, self-reported disease history, brain MRI-derived variables, and all-cause mortality in the LBC sets using a series of linear and logistic regression models, adjusting for age and sex (see Methods, Supplementary Table 4).

A small number of the explored associations were statistically significant (P_FDR_ < 0.05). Considering lifestyle and cognitive traits, the EpiScore was associated with smoking status in LBC1936 (standardized β = 0.117, P_FDR_ = 0.009) and with occupational social class in LBC1921 (β = −0.105, FDR *P* = 0.024). On the other hand, a higher self-reported alcohol consumption was associated with smoking status in LBC1921 (β = 0.17, P_FDR_ = 0.011). Considering disease history, the EpiScore was positively associated with high blood pressure in LBC1936 (OR_per SD of the EpiScore_ = 1.22, P_FDR_ = 0.012). Self-reported alcohol consumption was not significantly associated with any of the disease histories considered here. Further, we found a significant association between our EpiScore and time to all-cause mortality in LBC1936 (HR_per SD of the EpiScore_ = 1.16 [95% CI 1.05, 1.28]).

**Fig. 1:**
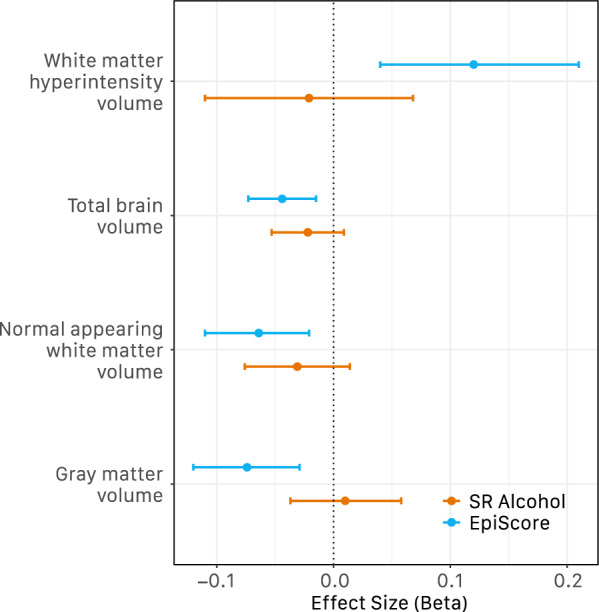
Self-reported (SR) alcohol consumption (units per week) and alcohol EpiScore associations with global brain imaging in LBC1936. Standardized effect sizes from age and sex-adjusted linear regression models shown along with 95% confidence intervals. Alcohol units defined as per NHS guidelines of 8 g/10 ml of pure ethanol

All brain MRI variables were found to be significantly associated with the EpiScore in LBC1936 but were not found to be significantly associated with self-reported alcohol consumption (Fig. 1). These included negative associations with total brain volume (β =  − 0.044, P_FDR_ = 0.012), grey matter volume (β =  − 0.074, P_FDR_ = 0.001), and normal-appearing white matter volume (β =  − 0.064, P_FDR_ = 0.012), and a positive association with white matter hyperintensity volume (β = 0.120, P_FDR_ = 0.012).

### Sex-specific EpiScore performance

Given the differences in average alcohol consumption between males and females, we explored sex-specific models (see Methods). However, the prediction performance, as measured by r and incremental *R*^2^ over a model accounting for age and sex, did not vary greatly across EpiScores (Supplementary Fig. 9, Supplementary Table 5) in both LBC1921 and LBC1936.

### Alcohol consumption variance explained by the methylome and EWAS

Next, we determined the proportion of variance in the alcohol consumption phenotype that can be explained by all CpG sites measured on a DNAm array (more specifically, the Illumina EPIC array, consisting of 752,722 CpGs after QC). To do this, we fitted a Bayesian sparse regression model and performed a variance partitioning analysis using BayesR+ (see Methods). BayesR+ has been shown to implicitly control for white cell proportions, which are typically estimated from the DNAm data, related participants, and other unknown confounders [26]. Three mixture distributions were specified, corresponding to possible small, medium, and large effect sizes for the CpGs (explaining 0.01%, 0.1% and 1% of the variance, respectively). We fit models using (1) “normal pattern” drinkers in Generation Scotland and (2) the full Generation Scotland cohort. Our analyses found that 45.0% (95% Credible Interval 39.7%, 50.5%) and 49.3% (95% Credible Interval 44.3%, 54.4%) of alcohol consumption (log(x + 1) transformed) were explained by all CpGs in models with “normal week” drinkers (those whose self-reported alcohol consumption was consistent with their normal drinking behaviour) and the full cohort, respectively.

**Fig. 2:**
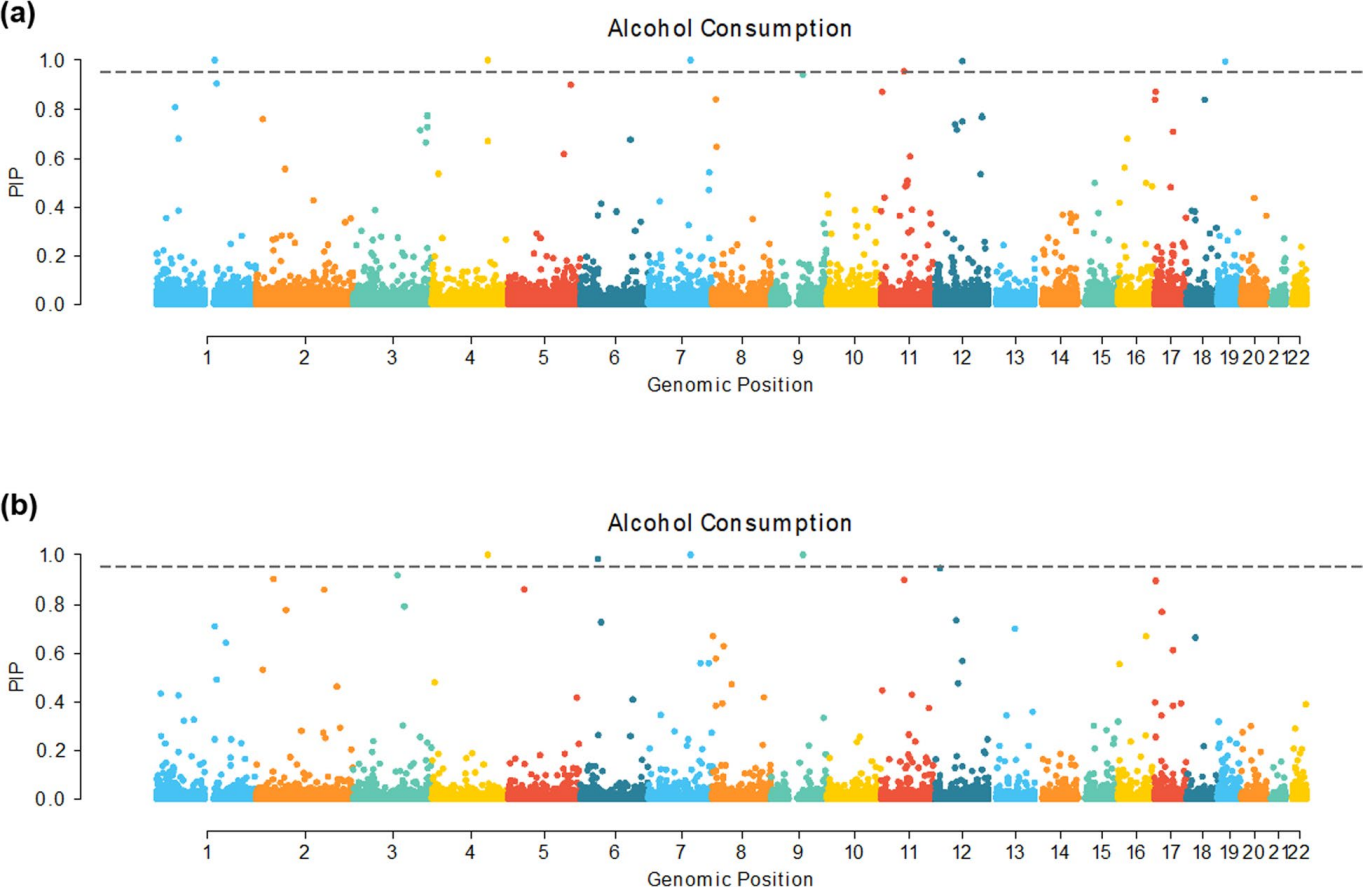
EWAS of alcohol consumption (units per week, log(x + 1) transformed) Manhattan plot. Model using 1) “normal week” drinkers and 2) full cohort. Threshold line set at posterior inclusion probability (PIP) = 0.95. Alcohol units defined as per NHS guidelines of 8 g/10 ml of pure ethanol

In addition to the variance components analysis, BayesR+ simultaneously conducts an epigenome-wide association study (EWAS—see Methods). This assesses the association between each CpG and the outcome, jointly across and conditionally on all possible CpGs. We found a total of four and six lead CpGs with a posterior inclusion probability (PIP) greater than 0.95 (Fig. 2, Table 4) in models considering just “normal week” drinkers and the full cohort, respectively. Two CpGs had a PIP greater than 0.95 in both models, and a total of eight unique lead CpGs were found.

**Table 4:** BayesR alcohol consumption (units per week, log(x + 1) transformed) EWAS associations with mean beta PIP > 0.8

Chr	CpG	Position	UCSC RefGene Name	Str	Mean Beta	SE	PIP	
4	cg06690548	139,162,808	SLC7A11	−	−0.0876	0.0005	1	Normal week drinkers
7	cg26774981	104,907,846	SRPK2;SRPK2;SRPK2	−	0.1039	0.0005	1	
9	cg05719472	80,914,400	PSAT1;PSAT1	−	0.0447	0.0003	0.999	
6	cg18120259	43,894,639	LOC100132354	+	−0.0910	0.0008	0.981	
1	cg24694018	145,457,621	POLR3GL	+	0.0572	0.0004	1	Everyone
4	cg06690548	139,162,808	SLC7A11	−	−0.0953	0.0004	1	
7	cg26774981	104,907,846	SRPK2;SRPK2;SRPK2	−	0.0660	0.0005	1	
2	cg06053623	68,778,314	–	−	−0.0464	0.0003	0.995	
9	cg03741185	18,201,510	IL12RB1	+	−0.0504	0.0004	0.994	
11	cg11376147	57,261,198	SLC43A1	−	−0.0798	0.0009	0.954	

Chr = chromosome; Str = strand. Alcohol units defined as per NHS guidelines of 8 g/10 ml of pure ethanol

We queried the EWAS catalog (accessed 14th May, 2024) for the eight aforementioned CpG sites and found that three had been previously linked to alcohol consumption, while one had been previously linked to alcohol withdrawal recovery (Supplementary Table 6). This search is not exhaustive, as not all studies deposit data in this resource. Indeed, six of the eight CpG sites were found in the largest previously published alcohol consumption EWAS making use of Generation Scotland data [11] (all but cg03741185 and cg06053623). Seven of the eight CpGs were found to be associated with at least one other trait in the EWAS catalog, including age, prevalent type 2 diabetes, serum high-density cholesterol, gestational age, serum triglycerides, blood pressure, BMI, and others.

Our lead CpGs mapped to the genes *POLR3GL, SLC7A11, SRPK2, PSAT1, IL12RB1, SLC43A1,* and *LOC100132354* (Table 4). *IL12RB1* has not been previously linked to alcohol consumption, based on EWAS Catalog output and the previously largest GS-based alcohol consumption EWAS.

## Discussion

Excessive alcohol consumption is one of the most important contributors to the global burden of disease, with important associations to conditions including cardiovascular disease, cancer, and more [3–5]. Alcohol has further been associated with DNAm differences via multiple mechanisms, [27] and as such, the altered methylome could offer clues into alcohol–disease links.

Here, we report a new alcohol EpiScore which explains up to 28% of the variance in self-reported consumption and performs similarly well across three UK-based and one US-based cohort. The score performs best in mid-to-older-aged adults and in those who stated that their consumption in the past week was reflective of a normal week, compared to those who drank less or more than normal. By contrast, the EpiScore showed poorest performance in the teenage subset of the ALSPAC cohort (correlation of 0.11). As highlighted by others [11, 28], our work suggests that an alcohol EpiScore is well placed to track chronic exposure. These findings also suggest that the relationship between alcohol and the methylome is dynamic and reversible. Indeed, a recent study found that a large number of CpG sites that were found to be associated with alcohol consumption presented differential methylation between former and current drinkers and found that alcohol-related hypomethylation is largely reversible upon cessation [16]. Our results suggest that changes to the methylome could be observed in short time frames, but longitudinal data with frequent time points would be needed to confirm this.

Our alcohol EpiScore also negatively associated brain MRI-derived measures (tested in the LBC1936 cohort), whereas self-reported alcohol consumption did not. Chronic alcohol use is associated with changes in brain structure and connectivity [29], and previous studies have reported links between higher alcohol consumption and lower white and grey matter volume [30], as well as with higher white matter hyperintensity volume [31]. A recent study making use of the UK Biobank brain MRI data (*N* = 36,585) [2] found that self-reported alcohol consumption was associated negatively and slightly non-linearly with both white matter and grey matter volumes, after accounting for covariates including age, sex, and BMI. There, consuming as few as 1–2 alcoholic drinks daily was associated with lower brain volume.

Previous studies have found that CpG pre-filtering ahead of elastic net greatly improves predictor performance when using this training method [32]. Our current results echo this, with an increase in prediction accuracy found when training on CpGs with a previously established association to alcohol consumption. This could be due to technical limitations of penalized regression when the number of predictors is much larger than the number of observations [33], alongside the screening out of CpGs with low intra-sample variability due to technical variance [34, 35]. We also show the importance of sample size when training EpiScores; compared to a previous EpiScore, trained in 2,819 unrelated Generation Scotland volunteers with “normal week” drinking patterns [8], our new score explained 1.7- and 2.8-fold more variance in a self-reported alcohol consumption phenotype from the Lothian Birth Cohorts of 1936 and 1921, respectively.

Alcohol consumption patterns and alcohol-related complications differ between the sexes [30, 36, 37]. In addition, sex differences in the methylome have been described [38]. However, we found that sex-specific EpiScores yielded very similar results when matching sample sizes and comparing to sex-agnostic models. This suggests that despite there being differences in consumption patterns, the methylation response to alcohol is similar across males and females for an equivalent consumption of units.

Our alcohol EWAS identified 8 sentinel loci, which mapped to 7 unique genes. Three of the genes the CpGs map to, which have already been reported to be associated with alcohol consumption in previous EWAS efforts [9, 11, 16, 39], are part of the aminotransferase family (*SLC7A11, PSAT1,* and *SLC43A1*). Alcohol is known to disrupt protein metabolism and amino acid transport [40, 41] and *SLC7A11*’s role in the liver–brain axis in alcohol-related disease and potential as a future drug target has been described [11]. One of our strongest CpG associations (cg26774981) mapped to the *SRPK2* gene, a kinase that controls alternative splicing. A recent paper found the regulation of alternative splicing by SRPK2 is implicated in lipogenesis in humans with alcohol-associated liver disease, thus making it a potential drug target [42]. One of the seven genes mapping to the CpG loci, we identified has not been linked to alcohol consumption: a type I transmembrane protein of the haemopoietin receptor superfamily (*IL12RB1*). Future work is needed to replicate these findings and to understand their potential role in alcohol-mediated disease aetiology.

Our study has several limitations. Firstly, the majority of the Generation Scotland and Lothian Birth Cohorts are of White British ancestry, which could lead to biases and difficulty translating these results to other population. However, the EpiScore performed as well or better in two external cohorts of diverse age ranges (ALSPAC) and ancestries (Sister Study). Secondly, as has been discussed previously, this study is based on an imperfect phenotype. Indeed, self-reporting has its limitations, and further details regarding alcohol consumption, such as a breakdown of type of drink consumed (beer, wine, spirits) and pattern of consumption (e.g. binge versus light drinking alongside seasonal variations), could help further untangle its relationship with human health and the methylome. It is also unknown if EpiScores can capture lifetime drinking patterns or if there is a particular period in life where alcohol is particularly detrimental for health. These limitations surrounding the phenotypic measurement of alcohol consumption perhaps limit the interpretation of the EpiScore and question what aspects of drinking behaviour it is reflecting. Replication of the current findings, along with more extensive comparisons with other health and lifestyle traits, may help to determine what aspects of alcohol consumption the EpiScore captures and where it might (and might not) be valid as a phenotypic proxy. Thirdly, DNAm was measured in whole blood, and therefore, these results may not apply to all blood cell types or other mechanistically relevant tissues such as brain. Finally, there were relatively subtle differences between the performance of various EpiScores during the feature pre-selection and population subsetting of the training dataset. Defining a “best” score is non-trivial, especially given that sample sizes varied between the whole cohort and those who reported drinking a similar amount to a normal week. Here, we selected the EpiScore with the highest point estimate values for *r* and *R*^2^ across the Generation Scotland test set for application in the external cohorts.

Going forwards, phenome-wide association studies of self-reported alcohol consumption and its EpiScore in relation to incident disease outcomes and health-related traits would help to quantify the utility of the latter in risk prediction settings. Running these analyses across diverse cohorts would also help to identify consistent trends versus those that might be bespoke to a particular study. Further, the addition of other objective biomarkers, such as blood alcohol, would further help to identify patterns of short-term versus long-term behavioural patterns in relation to DNA methylation differences. This would also help one to determine the accuracy and degree of bias with self-reported measures. However, such data are rarely collected in epidemiological cohort studies.

Whereas existing biomarkers can accurately measure chronic/heavy alcohol exposure, our EpiScore offers an opportunity to track consumption across all levels of exposure. There is also evidence to suggest that it can capture novel associations with brain health, compared to self-report metrics. Finally, we show that the EpiScore generalizes well across cohorts of diverse ages and ancestries. Future studies should determine if our EpiScore can help studies to impute an alcohol phenotype where self-report data are not available.

## Methods

### Generation Scotland (GS)

#### Overview

Generation Scotland is a Scottish family-based study with over 24,000 participants recruited between 2006 and 2011 [43]. Participants were aged between 18 and 99 years at recruitment, with a mean age of 47.5 years (SD 14.9). After exclusions (Supplementary Fig. 1), a total of 16,717 participants (9758 females and 6959 males) had measured blood-based DNAm (see Methods) and self-reported alcohol consumption data available (Supplementary Table 1 and Table 1). The mean units consumed in the week prior to completing the questionnaire and blood draw were 10.9 (SD 12.7, Supplementary Figs. 2 and 3). A total of 10,506 (62.8%) participants reported that this number was reflective of their usual drinking pattern with 1622 and 3756 noting it was less or more than they typically drink in a week (response unknown for *N* = 833).

#### DNA methylation

DNA methylation in blood at baseline (recruitment) was quantified for 18,413 Generation Scotland participants across three separate sets (N_Set1_ = 5087, N_Set2_ = 4450, N_Set3_ = 8876) using the Illumina MethylationEPIC (850 K) array. Individuals in Set 1 included a mixture of related and unrelated individuals. Set 2 comprised individuals unrelated to each other and to those in Set 1. Set 3 contained a mix of related individuals—both to each other and to those in Sets 1 and 2—and included all remaining samples available for analysis. Methylation data were processed across 121 experimental batches (N_Batches. Set1_ = 31, N_Batches, Set2_ = 30, N_Batches, Set3_ = 60).

Quality control details have been reported previously [44, 45]. Briefly, probes were removed based on (1) outliers from visual inspection of the log median intensity of the methylated versus unmethylated signal per array, (2) a bead count < 3 in more than 5% of samples, (3) ≥ 5% of samples having a detection *p*-value > 0.05, (4) if they pertained to the sex chromosomes, (5) if they overlapped with SNPs, and/or (6) if present in potential cross-hybridizing locations [46]. Samples were removed (1) if there was a mismatch between their predicted sex and recorded sex, (2) if ≥ 1% of CpGs had a detection *p*-value > 0.05, (3) if sample was not blood based, and/or (4) if participant responded “yes” to all self-reported diseases in questionnaires. A total of 752,722 CpGs remained after QC. Missing values were imputed using the mean of each CpG across all samples. Dasen normalization [47] was performed across all individuals.

#### Alcohol consumption data

Self-reported alcohol consumption was measured at baseline via questionnaires to obtain the number of units consumed in previous week (unit definition as per UK National Health Service: 8 g/10 ml of pure alcohol). Participants were also asked whether this was their usual drinking amount, or whether they had consumed more or less than normal. A total of 16,717 individuals had non-missing alcohol consumption data and methylation data—the rest of participants were excluded from this study (after exclusion, N_Set1_ = 4576, N_Set2_ = 4108, and N_Set3_ = 8033 individuals were left in sets 1, 2 and 3, respectively). Of these, 10,506 marked this quantity as representative of their typical weekly consumption, and 3756 stated this quantity was more than normal and 1622 less than normal (Supplementary Table 1, Supplementary Fig. 1).

### Lothian Birth Cohorts of 1921 and 1936 (LBC1921 and LBC1936)

#### Overview

LBC1921 and LBC1936 are longitudinal studies of ageing on individuals born in 1921 and 1936, respectively [20]. Study participants completed the Scottish Mental Surveys of 1932 and 1947 at approximately age 11 years old and were living in the Lothian area of Scotland at the time of recruitment in later life.

#### DNA methylation

Blood samples considered here were collected at around age 79 for LBC1921 and at around age 70 for LBC1936. DNA methylation was quantified using the Illumina HumanMethylation450K array, for a total of 692 (up to 3 repeated measurements from 469 individuals) and 2796 (up to 4 repeated measurements from 1043 individuals) samples from LBC1921 and LBC1936, respectively. Quality control details have been reported previously [48]. Briefly, probes were removed (1) if they presented a low (< 95%) detection rate with *p-*value < 0.01 and/or (2) if they presented inadequate hybridization, bisulphite conversion, nucleotide extension, or staining signal, as assessed by manual inspection. Samples were removed (1) if they presented a low call rate (< 450,000 probes detected at *p*-value < 0.01) and/or (2) if predicted sex did not match reported sex.

#### Self-reported alcohol consumption

Participants were asked about their usual alcohol consumption, including number of times alcohol is consumed per week, normal alcohol consumption, typical drink of choice, and glasses/pints consumed on average. From this information, alcohol consumption in units consumed per week was derived. A total of 436 and 895 individuals had non-missing alcohol consumption and methylome data available in LBC1921 and LBC1936 baseline, respectively, and were considered in this study.

### ALSPAC

#### Overview

The Avon Longitudinal Study of Parents and Children (ALSPAC) is a cohort study of pregnant women resident in Avon, UK, with expected dates of delivery between 1 April 1991 and 31 December 1992 [22, 23]. Among these, 20,248 pregnancies were identified as being eligible and the initial number of pregnancies enrolled was 14,541 resulting in 14,062 live births and 13,988 children who were alive at 1 year of age. At the start of the study, mothers invited their partners to complete questionnaires. In total, 121,113 partners have provided data and 3807 are currently formally enrolled. As part of Accessible Resource for Integrated Epigenomic Studies (ARIES) [49, 50], a subsample ALSPAC children, mothers and partners had DNAm assayed using the Illumina Infinium HumanMethylation450 or MethylationEPIC Beadchip array from peripheral blood samples collected at multiple time points from birth to middle age. The present study used DNAm measured from peripheral blood samples collected from ALSPAC children at ages 15–17 (time point “15up”) and 24 (time point “F24”) [51], and from ALSPAC mothers and partners [52] 18 years after the study pregnancy. Study data were collected and managed using REDCap electronic data capture tools hosted at the University of Bristol. REDCap (Research Electronic Data Capture) is a secure, web-based software platform designed to support data capture for research studies [53]. Please note that the study website contains details of all the data that are available through a fully searchable data dictionary and variable search tool (http://www.bristol.ac.uk/alspac/researchers/our-data/).

#### DNA methylation

Illumina Infinium HumanMethylation450 and MethylationEPIC Beadchip arrays were used to assess genome-wide DNAm patterns in peripheral blood. Samples across different time points were distributed in a semi-random manner across slides in order to mitigate batch effects. Data pre-processing and normalization were performed using the R package meffil as previously described [50]. Samples with large numbers of undetected probe signals were removed, along with those that had sex or genotype mismatches. Probes undetected in more than 20% of samples were excluded.

#### Self-reported alcohol consumption

Alcohol consumption was measured as the estimated number of units consumed on average during the week the year before blood sample collection for DNAm analysis. Consumption was estimated multiplying alcohol intake frequency per week by intake quantity. Frequency was assessed by the question “How often do you have a drink containing alcohol”, with possible responses including “Never”, “Monthly or less”, “2–4 times a month”, “2–4 times a week”, and “4 or more times a week”. “Never” drinking respondents were considered non-drinkers and were included in all primary analyses. Quantity was assessed by asking the number of drinks consumed where “one drink referred to ½pint of beer/cider, a small (125 ml) glass of wine or a single (25 ml) measure of spirit”, each of which is roughly equivalent to one UK alcohol unit (8 g of ethanol).

### Sister study

#### Overview

The Sister Study is a US-nationwide prospective cohort study of 50,884 women enrolled between 2003 and 2009; women were eligible for enrolment if they resided in the USA and were breast cancer-free themselves but had a biological sister who was previously diagnosed. As part of study enrolment when all women were breast cancer-free, women completed self-reported questionnaires and an in-home visit where a whole blood sample was collected. Information about obtaining data from the Sister Study can be found at: https://sisterstudy.niehs.nih.gov/English/coll-data.htm.

#### DNA methylation

Two case–cohort samples of women were selected for DNAm profiling. In 2014, blood DNA samples from 2878 self-identified non-Hispanic White women were assayed on the Infinium HumanMethylation450 BeadChip [54]. This sample included 1542 women who were diagnosed with breast cancer in the years following enrolment (mean time to diagnosis: 4 years). In 2019, blood DNA samples from 2599 self-identified Black (Hispanic and non-Hispanic) and non-Hispanic White women were assayed on the Infinium MethylationEPIC v1 BeadChip [55]. This sample included 999 women who were diagnosed with breast cancer in the years following enrolment (mean time to diagnosis: 5 years). Self-identified Hispanic and non-Hispanic Black women were over-sampled for DNAm profiling in order to maximize the racial and ethnic diversity of the MethylationEPIC sample.

For both DNAm samples, DNAm data were preprocessed using the *ENmix* software pipeline, which included background correction, dye bias correction, inter-array normalization, and probe-type bias correlation [56–58]. Samples were excluded if they did not meet quality control measure including bisulphate intensity < 4000, had greater than 5% of probes with low-quality methylation values (detection *P* > 0.000001, < 3 beads, or values outside 3 times the interquartile range), or were outliers for their methylation beta value distributions. In total, 178 participants from the HumanMethylation450 sample and 250 participants from the MethylationEPIC sample were excluded for not meeting quality control measures.

#### Alcohol consumption

Participants’ history of alcohol consumption was obtained within 1 year of blood draw as part of a baseline questionnaire for alcohol use. Women reported information including the age at which they started and stopped drinking alcohol. The frequency of alcohol consumption was reported as days per week, month, or year by decade of life. The alcohol use variable used in this study was a derived variable that represented the average number of drinks per week over the last twelve months.

### EpiScore of alcohol consumption: who to train on, and how?

In an effort to assess the optimal cohort sample and feature space to train on, multiple EpiScores were assessed. The Generation Scotland cohort was divided into a training (sets 1 and 2, N = 8684) and a testing dataset (set 3, N = 8033). EpiScores were trained on the full training dataset, as well as just on the “normal week” drinkers (N = 5618). Further, EpiScores were trained on the full methylome (386,399 CpGs after limiting measured features to those also present in the Illumina 450 K array for wider applicability) or on a subset of 3999 epigenome-wide significant CpGs (*P* < 3.6 × 10^−8^) that have been previously linked to alcohol consumption in three separate studies not using Generation Scotland [9, 16, 25].

Elastic net penalized regression was used to train our EpiScores on log-transformed alcohol consumption + 1 (*glmnet* package in R, v4.1). CpG beta values in the training set were scaled to mean zero and unit variance ahead of elastic net, thus obtaining standardized regression effect sizes. The *L*_1_, *L*_2_ mixing parameter was set at α = 0.5, and tenfold cross-validation was performed to select the shrinkage parameter (λ) that minimized the mean cross-validated prediction error.

Predictive performance for each EpiScore was assessed by projecting the latter into the testing dataset by multiplying each CpG by its estimated weight and performing summation, scaling CpG beta values beforehand to mean zero and unit variance. Pearson correlation (r) of the EpiScore with measured log alcohol consumption + 1, as well as the incremental *R*^2^ upon the addition of the EpiScore to a linear regression model adjusting for age and sex, was then calculated. EpiScore statistical significance was assessed considering the marginal test for the beta in the linear regression model adjusting for age and sex (assessing whether beta is significantly different from zero).

### Training the EpiScore in generation Scotland and testing in the Lothian Birth Cohorts, ALSPAC, and sister study

Having established that training on all individuals with self-reported alcohol consumption data (regardless of whether this pattern reflected a typical week or was more or less than normal), and on a pre-filtered set of CpGs, yields the better performing EpiScore, we next trained on the full Generation Scotland cohort (N = 16,717). As with the creation of previous EpiScores, elastic net penalized regression was used with α = 0.5 and tenfold CV. This EpiScore was then projected and tested on the Lothian Birth Cohorts of 1921 and 1936, ALSPAC, and the Sister Study. Its performance was again assessed via a Pearson correlation with self-reported alcohol consumption (log(x + 1) transformed) and the incremental R^2^ upon the addition of the EpiScore to a linear regression model adjusting for age and sex.

#### Sex-specific EpiScores

Sex-specific EpiScores were trained after matching sample sizes (thus ensuring larger sample sizes weren’t driving better prediction). Given that the smallest sex-stratified sample size was N = 6958 (males), we trained male-specific EpiScore on the full male sample set, a female-specific EpiScore trained on a random subsample of 6958 female participants, and a sex-agnostic EpiScore trained on equal numbers of males and females with overall sample size also being 6958 (N_F_ = 3479, N_M_ = 3479).

To assess performance, using the same metrics and testing LBC dataset described previously, we tested the resulting EpiScores in three different ways: (1) a sex-specific manner by which predictions are obtained using each testing sample’s sex-specific EpiScore, (2) an opposite-sex manner, by which the EpiScore trained on the opposite sex of the testing sample is used to obtain predictions, and (3) a sex-agnostic manner, by which all samples, regardless of sex, are predicted using the EpiScore trained on both males and females.

### EpiScore and self-reported alcohol consumption associations in the Lothian Birth Cohorts

Associations between multiple phenotypes and self-reported alcohol consumption, as well as with our generated EpiScore trained on the full Generation Scotland cohort, were evaluated separately in LBC1921 and LBC1936. For each phenotype, linear regression models were run, adjusting for age, sex, and either self-reported alcohol consumption or the epigenetic predictor. Phenotypes considered included body mass index (BMI in kg/m^2^), hand grip strength (maximum of left and right hand measurements, in kg), self-reported years of education, self-reported smoking status (never smoker, ex-smoker, and current smoker), number of smoked packs per day, measured time taken to walk 6 m (in seconds), occupation-based social class (measured as social grades based on highest reached occupation [59]), and depression and anxiety scores (HADS-D and HADS-A total from the Hospital Anxiety and Depression questionnaire [60]). Associations with blood biomarkers cholesterol and triglycerides were also assessed.

Self-reported alcohol and alcohol EpiScore associations with self-reported prevalent disease were evaluated using logistic regression, adjusting for age and sex. These included CVD, stroke, neoplasm, high blood pressure, diabetes, and thyroid dysfunction. Associations with time to all-cause mortality were assessed using a Cox proportional hazards model with age and sex as covariates, using the *survival* R package (v3.5), with time to all-cause mortality or censoring as the survival outcome.

Finally, associations with multiple brain imaging phenotypes measured in LBC1936 were considered. Briefly, structural and diffusion tensor (DTI) MRI acquisition and processing in LBC1936 were performed at Wave 2 (age 73 years) according to an open-access protocol [61]. Total brain, grey matter and normal-appearing white matter (NAWM) volumes were calculated using a semi-automated multi-spectral fusion method [62]. Intracranial volume was determined semi-automatically using Analyze 11.0™. Total brain, grey matter, and white matter volume measurements were scaled to mean zero and unit variance, and associations with self-reported alcohol consumption and the alcohol EpiScore were assessed via linear regression, adjusting for age, sex, and intracranial volume.

Sample sizes varied for each phenotype considered given missing values arising from incomplete participant questionnaires (Supplementary Table 3). Association *P*-values were FDR corrected (using the Benjamini–Hochberg procedure) to account for multiple testing within each LBC cohort.

### Variance components analysis and EWAS using BayesR+ 

BayesR+  [26], a software implementation of a Bayesian regression modelling framework, which implicitly controls for white cell proportions, related participants, and other unknown confounders, was used to estimate the variance accounted for in alcohol consumption by methylation data, as well as estimate its association with each measured CpG (a total of 752,722). To remove the effects of age, sex, and smoking (via an EpiScore [7]), the input for BayesR+ was defined by the residuals of a linear regression model for alcohol consumption (log(x + 1) transformed) with those variables as covariates. CpG M-values were pre-corrected in a similar way, regressing out age, sex, smoking EpiScore, and batch. They were subsequently scaled to have mean zero and unit variance.

Full details of the BayesR+ modelling framework have been previously described [26]. Briefly, BayesR+ utilizes Gibbs sampling to generate draws from the posterior distribution conditional on the input data, setting prior mixture variances to 0.0001, 0.001 and 0.01, corresponding to possible small, medium, and large effect sizes of the CpGs considered (explaining 0.01%, 0.1% and 1% of the variance of the phenotype of interest, respectively). After a burn-in of 5000 draws, 10,000 draws were retained. Subsequently, a thinning of five draws was applied to reduce autocorrelation (i.e. 1000 iterations are used when reporting results for this analysis). The convergence of the hyperparameters was evaluated through the Geweke test [63], as well as assessing parameter values across iterations and assessing autocorrelation. For each probe, the proportion of iterations for which the probe was categorized as having a nonzero effect was calculated, this yielding the posterior inclusion probability (PIP). A PIP value over 0.95 (95%) was deemed to reflect an epigenome-wide significant CpG locus.

Variance components were estimated by the mean sum of squared standardized posterior effect sizes across the 1000 iterations. Individual effect sizes were estimated as the average across the 1000 iterations for each CpG. Models were run considering data for the full Generation Scotland cohort, as well as just the subset of “normal pattern” drinkers.

## Supplementary Information

Additional file1 (XLSX 51 KB)

Additional file2 (DOCX 9141 KB)

## Data Availability

According to the terms of consent for Generation Scotland participants, access to data must be reviewed by the Generation Scotland Access Committee. Applications should be made to access@generationscotland.org. Lothian Birth Cohort data are available on request from the Lothian Birth Cohort Study, University of Edinburgh (https://www.ed.ac.uk/lothian-birth-cohorts/data-access-collaboration). Lothian Birth Cohort data are not publicly available due to them containing information that could compromise participant consent and confidentiality. ALSPAC data are available on request from bona fide researchers. The study website contains details of all the data that are available through a fully searchable data dictionary and variable search tool (http://www.bristol.ac.uk/alspac/researchers/our-data/). Data from the Sister Study are available upon request via the Sister Study website (https://sisterstudy.niehs.nih.gov/English/coll-data.htm.) All custom R (version 4.3.0), Python (version 3.9.7), and bash code is available with open access at the following GitHub repository: https://github.com/elenabernabeu/methylomics_alcohol. EWAS summary statistics are available via  Edinburgh DataShare: https://datashare.ed.ac.uk/handle/10283/8929.
