# Supplementary material for: Blood-based epigenome-wide association study and prediction of alcohol consumption
Source: Clin Epigenetics. 2025 Jan 25;17:14. doi: 10.1186/s13148-025-01818-y (PMC11762500; doi:10.1186/s13148-025-01818-y)
Supplement: Supplementary file 2 — Additional file2 (DOCX 9141 KB) [file 13148_2025_1818_MOESM2_ESM.docx]

**Blood-based DNA methylation study of alcohol consumption**

**Supplementary Figures**

**
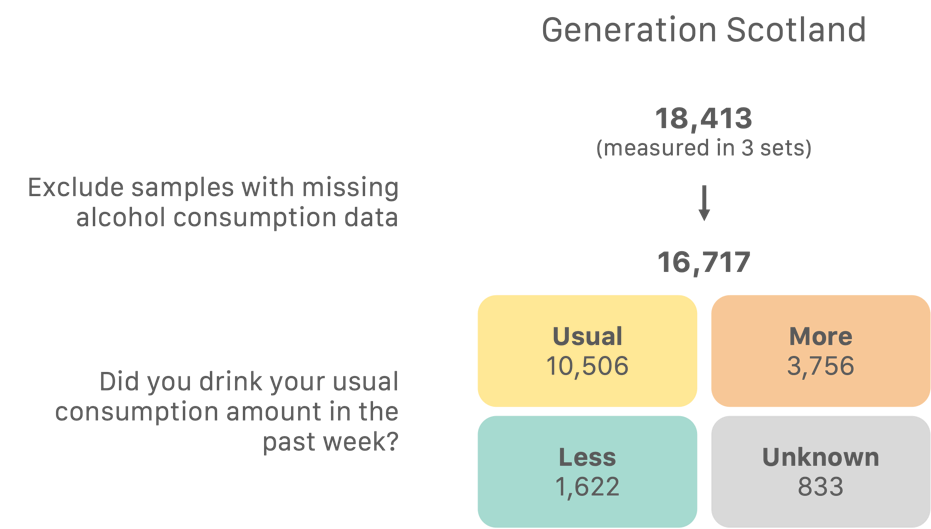
**

**Figure S1**. Generation Scotland sample breakdown.

a)


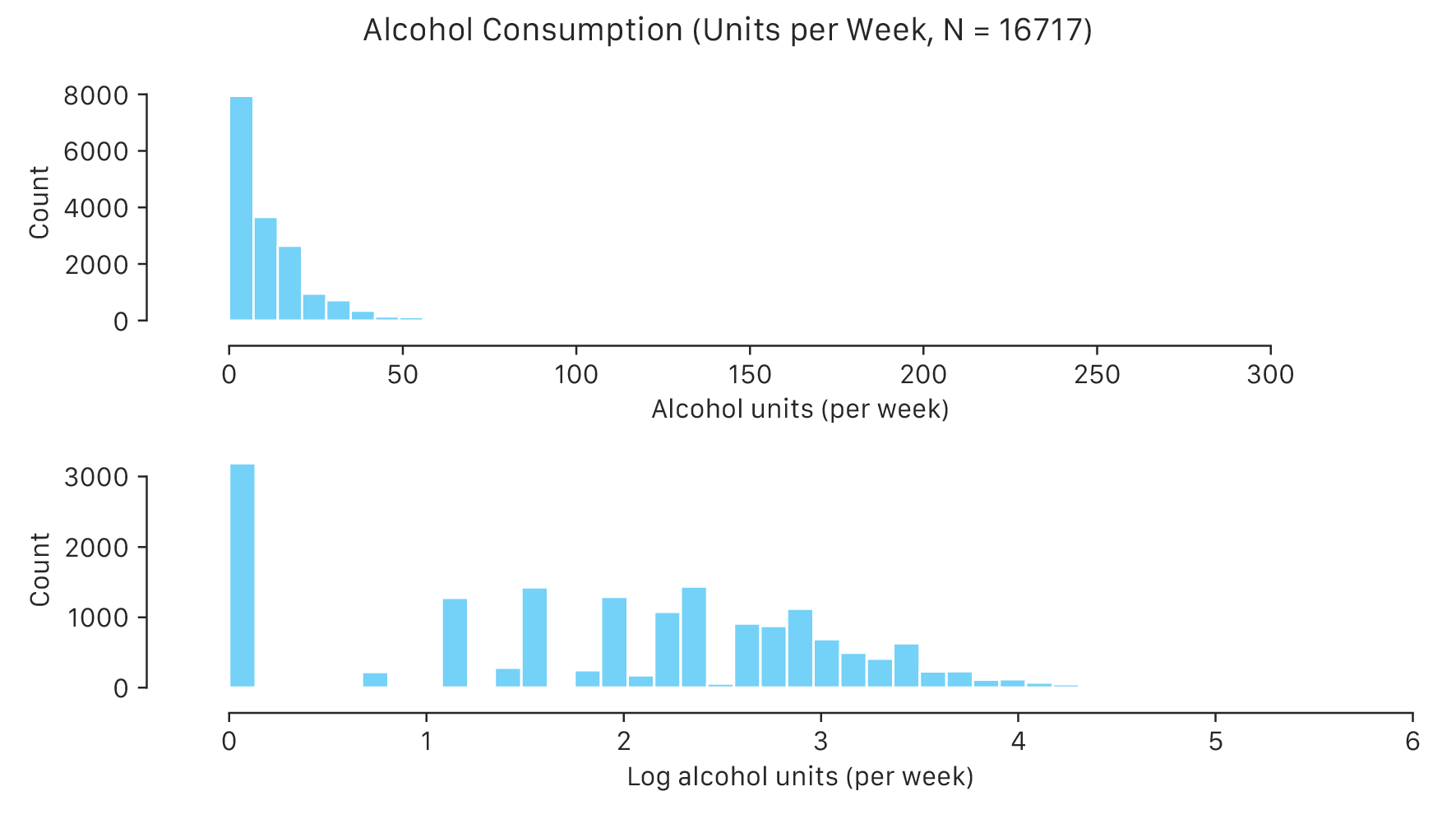


b)


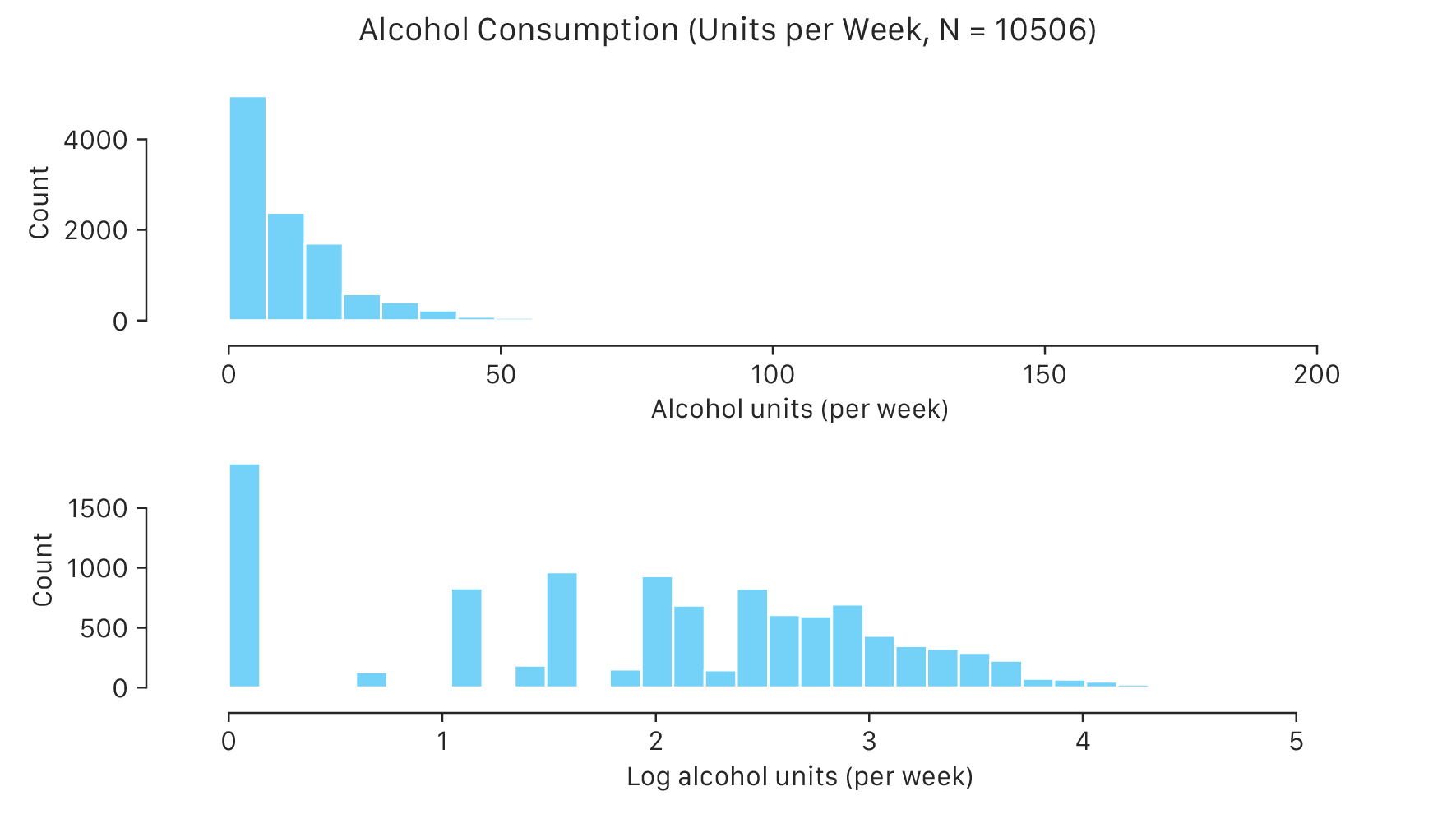


**Figure S2**. Histogram of alcohol units (measured and in log scale +1; unit = 8g/10ml of pure alcohol as per NHS definition) consumed in the previous week, for a) everyone in Generation Scotland and b) just “normal week” drinkers.

a)


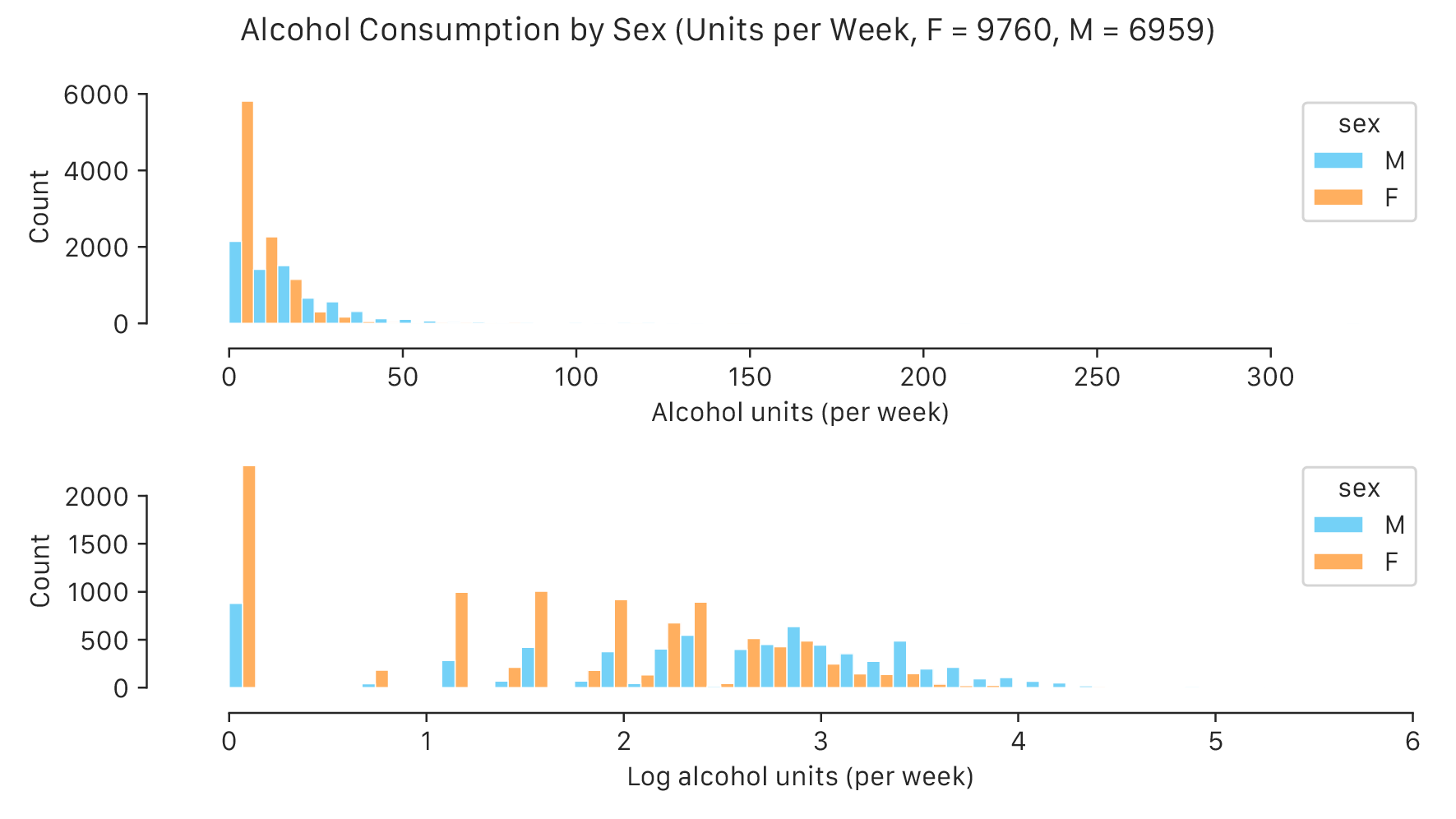


b)


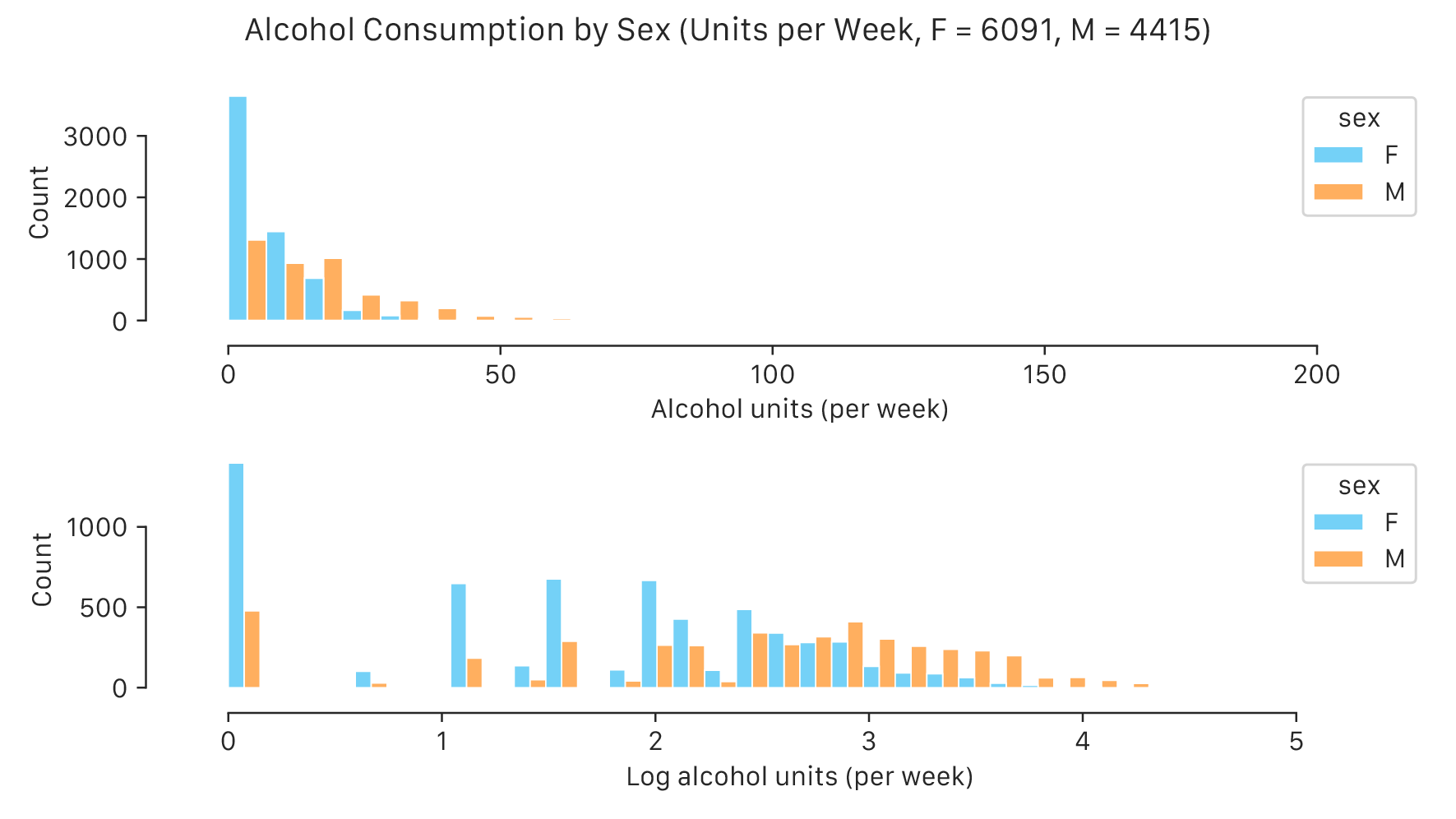


**Figure S3**. Histogram of alcohol units (measured and in log scale +1; unit = 8g/10ml of pure alcohol as per NHS definition) consumed in the previous week, stratified by sex, for a) everyone in Generation Scotland and b) just “normal week” drinkers.

**Figure S4**. **Measured alcohol consumption against 4 EpiScores of alcohol consumption, trained and tested in different subsets of Generation Scotland.** Alcohol consumption (units per week; unit = 8g/10ml of pure alcohol as per NHS definition) shown in log(x+1)-scale. EpiScores trained on everyone in the training set in Generation Scotland, just on “normal week”/”usual” drinkers in the training set, and filtering CpGs or not. Pearson correlations between the two measures indicated. Colour indicates density of points, with white/orange symbolising higher density.

1. “Normal week”/”usual” drinkers

1. “Normal week”/”usual” drinkers + filtered

1. Everyone

1. Everyone + filtered

**Figure S5. Measured alcohol against 4 alcohol consumption EpiScores.** Alcohol consumption (units per week; unit = 8g/10ml of pure alcohol as per NHS definition) shown in log(x+1)-scale. EpiScore trained in subset of Generation Scotland (either everyone or “normal week” drinkers), and tested in unused subset. Prediction performance stratified by category of testing sample (“normal week” drinker, drank more than usual in measured week, or drank less than usual in measured week). a) Trained on “normal week” drinkers, using all CpGs, b) trained on “normal week” drinkers using filtered CpGs, c) trained on everyone, using all CpGs, d) trained on everyone using filtered CpGs.

**Figure S6**. EpiScore performance in LBC1921 and LBC1936 for EpiScore trained in full Generation Scotland cohort. Alcohol consumption (units per week; unit = 8g/10ml of pure alcohol as per NHS definition) in log(x+1)-scale. Colour indicates density of points, with white/orange symbolising higher density.

**Figure S7**. EpiScore performance across five ALSPAC cohorts. Alcohol consumption (units per week; unit = 8g/10ml of pure alcohol as per NHS definition) in log(x+1)-scale. Colour indicates density of points, with white/orange symbolising higher density.

**Figure S8**. EpiScore performance across two Sister Study cohorts. Alcohol consumption (units per week) in log(x+1)-scale. Colour indicates density of points, with white/orange symbolising higher density. “Units per week” was a derived variable that represented the average number of drinks per week over the last year.

**Figure S9. Sex-specific EpiScore performance test in the Lothian Birth Cohorts.** Measured alcohol consumption (units per week; unit = 8g/10ml of pure alcohol as per NHS definition) is compared against three different EpiScore modalities (sex-agnostic, same-sex, and opposite-sex) in the Lothian Birth Cohorts. Alcohol consumption in log(x+1)-scale.
